# Research on simulation of permanent magnet synchronous motor in full speed range

**DOI:** 10.1371/journal.pone.0320786

**Published:** 2025-04-21

**Authors:** Mingrun Yuan, Yuke Meng, Xiaozhong Qi, Xiao Li, Ning Zhang

**Affiliations:** 1 College of Information and Electronic Technology, Jiamusi University, Jiamusi, Heilongjiang, China; 2 College of Automation, Chongqing University, Chongqing, China; 3 College of Mechanical Engineering, Jiamusi University, Jiamusi, Heilongjiang, China; SRM Institute of Science and Technology (Deemed to be University), INDIA

## Abstract

This paper studies the speed regulation simulation of permanent magnet synchronous motor. First, this paper analyzes the mathematical model of permanent magnet synchronous motor, and studies the control strategy of Id = 0, maximum torque-current ratio and weak magnetic leading Angle under synchronous rotation coordinate system. Secondly, the control strategy model is established on MATLAB/SIMULINK platform, including coordinate transformation, space vector pulse width modulation (SVPWM), proportional integration regulator, three-phase inverter, MTPA, weak magnetic core control algorithm module. Meanwhile, an APP for real-time monitoring of motor simulation model operation, control and parameter adjustment is designed by using APP Designer toolbox. Finally, the start and stop, speed increase and decrease, load surge torque and motor high-speed operation of the electric motorcycle PMSM are simulated in the actual operation. The experimental results show that the system has strong response ability and anti-interference ability under the control of current and speed PI regulator. In the motor start-stop state, the MTPA control strategy can distribute large electromagnetic torque during start-up, effectively improve the efficiency of the inverter and save costs. Compared with MTPA and Id = 0 control strategies, weak magnetic control has excellent speed increase effect, up to 6000r/min, and has strong anti-interference ability. Through the control method of the leading Angle, the dynamic switching between MTPA and weak magnetic control strategy is realized, and the running speed range of PMSM is effectively extended.

## Introduction

Currently, China is vigorously developing the new energy electric vehicle industry. Electric motorcycles, due to their environmental benefits, high speed, and excellent acceleration, have attracted strong market attention. The Ministry of Industry and Information Technology of China has issued policies requiring the cessation of production and sale of fuel vehicles. Electric motorcycles powered by new energy have rapidly developed in the Chinese market. Data shows that in April, the production and sales of new energy electric motorcycles in China were 1.7626 million and 1.7541 million units, respectively, with year-on-year increases of 2.36% and 6.75%. Today, China’s new energy electric motorcycles have established a leading global trend [1]. Electric motorcycles use new energy batteries as their energy source, converting electrical energy into mechanical energy through a motor to propel the vehicle forward. In this context, the performance of new energy electric motorcycles is strongly related to the motor and controller [2,3]. With the widespread and rapid development of rare earth materials in China, the market for permanent magnet synchronous motors is also expanding [4]. Scholars both domestically and internationally are researching motor speed control, significantly improving the performance of permanent magnet synchronous motor (PMSM) speed control systems. PMSMs are known for their fast response, high power density, high low-speed torque density, high efficiency, and high reliability [5].

With the advancement of information electronics and power electronics technology, rotor flux-oriented control systems are also developing rapidly. Motor control based on Field-Oriented Control (FOC) has also progressed quickly, with current mainstream control schemes including Id = 0, Maximum Torque Per Ampere (MTPA), and advanced angle weak magnetic control [6,7]. Reference [8] proposes an improved scheme for speed-current PI double-loop control, introducing voltage feedforward decoupling to enhance steady-state performance. Reference [9] employs basic FOC speed-current loop control, but this scheme does not significantly improve the performance or speed control range of PMSMs. Reference [10] addresses the voltage limitations of three-phase inverters, proposing MTPA control below base speed and weak magnetic control above base speed to improve motor operating efficiency. Reference [11] proposes a sliding mode observer-based error feedback strategy for large torque pulsations but does not achieve the desired results for the speed control range of PMSMs.

To maximize the utilization of magnetic reluctance torque, many scholars have studied the MTPA strategy. Main computational methods include direct formula calculation, two-dimensional lookup tables, and MTPA trajectory search. Reference [12] introduces a fitting solution method for MTPA online operation, replacing the traditional two-dimensional lookup table. Reference [13] presents a high-performance torque control scheme for PMSMs, focusing on steady-state and transient torque dynamics under maximum torque current conditions, achieving precise, efficient, and robust torque control. Reference [14] normalizes the complex fourth-order equation of MTPA and uses curve fitting for approximation, simplifying the complexity of MTPA calculations. Reference [15] addresses the large computational burden of traditional PMSM weak magnetic control strategies, which involve various high-order computations, by using an advanced angle weak magnetic control method. This method redistributes stator currents Id and Iq through advanced angle and trigonometric calculations, allowing smooth switching between MTPA and weak magnetic control. Reference [16] proposes a hybrid control combining MTPA, advanced angle weak magnetic control, and MTPV deep weak magnetic control to achieve stable transitions across different speed regions (above and below base speed) and addresses stability in the deep weak magnetic region of PMSMs. Reference [17] highlights the significant impact of torque-speed relationships on motor control accuracy, proposing a simulation study of weak magnetic control for vehicle permanent magnet brushless DC motors. This study tests motor operating efficiency and stability, verifying the feasibility of simulation results under different operating conditions.

This project aims to simulate and design a high-performance, low-cost electric motorcycle controller to implement efficient weak magnetic control technology for permanent magnet synchronous motors, enhancing the efficiency and performance of electric motorcycles. First, optimizing the drive control system can improve the power performance of electric vehicles, providing stronger acceleration and higher top speeds, thus enhancing user experience and market competitiveness. Second, it can increase the operating efficiency and range of electric vehicles. By accurately controlling motor speed and output power, energy loss can be minimized, battery utilization can be maximized, and the vehicle’s range can be extended.

Main work of this study:

1. Develop the mathematical model of a permanent magnet synchronous motor (PMSM) in the synchronous rotating coordinate system, including the motor’s electromagnetic dynamic equations, loss model, and torque model. Design control strategy algorithm models for Id = 0 control, maximum torque per ampere (MTPA), and flux-weakening control according to motor control requirements.
2. Build models for the three control strategies in Matlab, and design and simulate a high-performance, low-cost electric motorcycle controller to achieve efficient flux-weakening control for PMSM. Improve the efficiency and performance of electric motorcycles. Analyze and compare the strengths and weaknesses of the three strategies. Propose a control method based on dynamic working region optimization, which dynamically adjusts control strategy parameters to balance energy efficiency and torque output stability under different speeds and load conditions.
3. Design a dedicated APP-based visual operation interface for the controller, enabling bidirectional interaction between the APP and the Simulink electric motorcycle controller model. Use a graphical interface to display vehicle operating status, real-time energy consumption, and speed dynamic curves, while providing real-time monitoring of controller operating conditions and motor performance.

## Methods

The research object of this topic is the built-in permanent magnet synchronous motor (IPMSM), the application object is the electric motorcycle, and the research content is the control strategy of the base speed up and down of the permanent magnet synchronous motor. In order to simplify the study of the control strategy of PMSM, the following ideal motor assumptions are made.

(1) Ignore the saturation, eddy current and hysteresis loss of the motor core;
(2) Ignore rotor damping winding, permanent magnet damping effect;
(3) Ignoring the conductivity of rotor permanent magnet material;
(4) The stator three-phase winding current is a symmetrical three-phase sine wave current, and the permanent magnet only generates a sine wave air gap magnetic field, ignoring higher harmonics [18].

In order to facilitate subsequent PMSM algorithm research, synchronous rotation coordinate system is usually selected for modeling and analysis in PMSM mathematical model [18]. Therefore, the PMSM stator voltage equation under the d-q axis is:


$$\{\begin{array}{c} {\text{u}}_{\text{d}}{\text{=Ri}}_{\text{d}}\text{+}\frac{\text{d}}{\text{dt}}{\text{ψ}}_{\text{d}}{\text{-ω}}_{\text{e}}{\text{ψ}}_{\text{q}}\\ {\text{u}}_{\text{q}}{\text{=Ri}}_{\text{q}}\text{+}\frac{\text{d}}{\text{dt}}{\text{ψ}}_{\text{q}}{\text{+ω}}_{\text{e}}{\text{ψ}}_{\text{d}} \end{array}$$ (1)

The stator flux equation is:


$$\{\begin{array}{c} {\text{ψ}}_{\text{d}}{\text{=L}}_{\text{d}}{\text{i}}_{\text{d}}{\text{+ψ}}_{\text{f}}\\ {\text{ψ}}_{\text{q}}{\text{=L}}_{\text{q}}{\text{i}}_{\text{d}} \end{array}$$ (2)

By combining the above formula (1) and (2), the stator flux equation is inserted into the stator voltage equation, and the following formula can be obtained:


$$\{\begin{array}{c} {\text{u}}_{\text{d}}{\text{=Ri}}_{\text{d}}{\text{+L}}_{\text{d}}\frac{\text{d}}{\text{dt}}{\text{i}}_{\text{d}}{\text{-ω}}_{\text{e}}{\text{L}}_{\text{q}}{\text{i}}_{\text{q}}\\ {\text{u}}_{\text{q}}{\text{=Ri}}_{\text{q}}{\text{+L}}_{\text{q}}\frac{\text{d}}{\text{dt}}{\text{i}}_{\text{q}}{\text{+ω}}_{\text{e}}（{\text{L}}_{\text{d}}{\text{i}}_{\text{d}}{\text{+ψ}}_{\text{f}}） \end{array}$$ (3)

**Fig 1:**
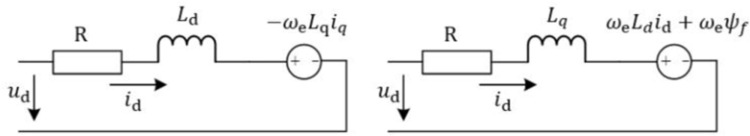
PMSM equivalent circuit diagram.


According to the above formula (3), it can be converted to the equivalent circuit diagram of the motor, as shown in Fig 1. PMSM is equivalent to d-q axis resistance, d-q axis inductance, and coupled electromotive force. In addition, we find that the mathematical model variables are decoupled and reduced in d-q coordinate system. The expression of electromagnetic torque under the d-q axis is as follows. The subsequent discussion of this topic is to study the mathematical model under this coordinate system.

$${\text{T}}_{\text{e}}\text{=}\frac{\text{3}}{\text{2}}{\text{p}}_{\text{n}}{\text{i}}_{\text{q}}\left[{\text{i}}_{\text{d}}\left({\text{L}}_{\text{d}}{\text{-L}}_{\text{q}}\right){\text{+ψ}}_{\text{f}}\right]$$ (4)

It can be seen from the PMSM structure[16] that the stator inductance of the surface mount motor structure meets Ld=Lq, the electromagnetic torque is proportional to the alternating axis current iq, in addition, pn, ψf are the number of poles of the motor and the flux linkage, and the characteristics of PMSM are constant.

The inductance of the stator in the built-in motor structure does not satisfy Ld = Lq, and the formula for electromagnetic torque is (4). In this project, a PMSM with a built-in motor structure is chosen to implement the speed control system for the permanent magnet synchronous motor of an electric motorcycle.

### Vector control algorithm

#### Vector control strategy analysis.

Vector control mainly draws on the principle of DC motor torque control and simplifies the PMSM complex control. The FOC splits the current vector into the direct axis id and the alternating axis iq components to simulate the torque control of a DC motor. By decoupling the current vector, the running performance and stability of PMSM are effectively improved.

**Fig 2:**
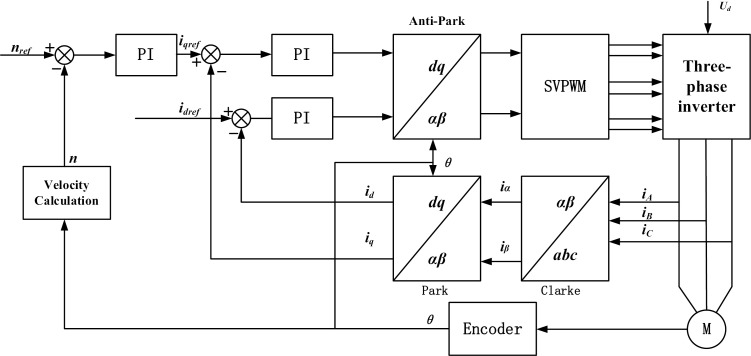
Vector control block diagram.

Fig 2 is the vector control block diagram, which mainly includes coordinate transformation module, space vector pulse width modulation module, three-phase inverter module, permanent magnet synchronous motor, speed current sensor module and speed and current PI controller module. The control process is as follows: First, the target speed of the motor is set, and then the target speed value is differentiated from the feedback value of the actual speed to obtain the input of the speed outer loop controller. Then, the speed outer loop controller converts the speed error into the target current signal, and differentiates the signal with the stator cross axis current and direct axis current feedback, and then acts as the input of the current inner loop controller. Then, the current inner loop controller outputs the voltage value, inverts the cross-axis and direct-axis voltage Park, and sends the result to the space vector pulse width modulation module (SVPWM). SVPWM outputs a three-phase complementary pulse-width modulation signal that acts on the three-phase inverter circuit to control the motor. The output current of the three-phase inverter is collected by the three-phase current sensor, converted through coordinate transformation (including Clark transformation and Park transformation), and used as the input of the current controller to achieve closed-loop control of the system.

### MTPA control algorithm

The maximum torque-current ratio (MTPA) is an optimal control method of current flow, which can realize the minimum and maximum proportional control of stator current and torque. MTPA can reduce the copper loss of the motor, which is conducive to the work of the inverter switching device, reduce the operating cost and improve the working efficiency of the motor. In addition, for the controller system of electric motorcycle, the inverter capacity is certain, and MTPA control can make the output current required by the inverter smaller and reduce the requirement on the power of the inverter [17].

The electromagnetic torque $T_{e}$ of PMSM consists of two parts: permanent magnet torque and magneto resistive torque. The permanent magnet torque is proportional to the stator current $i_{q}$. Magnetoresistive torque is the electromagnetic torque generated by the salient pole effect, proportional to the product of the stator alternating direct axis current, and related to the number of poles of the motor. The salient pole effect is caused by the asymmetry of the stator alternating direct axis magnetic circuit and the asymmetry of alternating axis inductance $L_{q}$ and direct axis inductance $L_{d}$.

Electromagnetic torque is completely controlled by permanent magnet torque and magneto resistive torque. The electromagnetic torque can be controlled by adjusting the alternating axis current and the direct axis current. The MTPA control strategy is to calculate the optimal configuration relationship between the alternating axis current and the direct axis current by giving the electromagnetic torque, so as to achieve the maximum torque output under the same current consumption.

By transforming Equation (4), the relationship between the quadrature-axis current $i_{q}$, electromagnetic torque $T_{e}$, and the direct-axis current can be derived.


$${\text{i}}_{\text{q}}\text{=}\frac{{\text{2T}}_{\text{e}}}{{\text{3n}}_{\text{p}}{\text{ψ}}_{\text{f}}{\text{+3n}}_{\text{p}}\left({\text{L}}_{\text{d}}{\text{-L}}_{\text{q}}\right)\cdot {\text{i}}_{\text{d}}}$$ (5)

**Fig 3:**
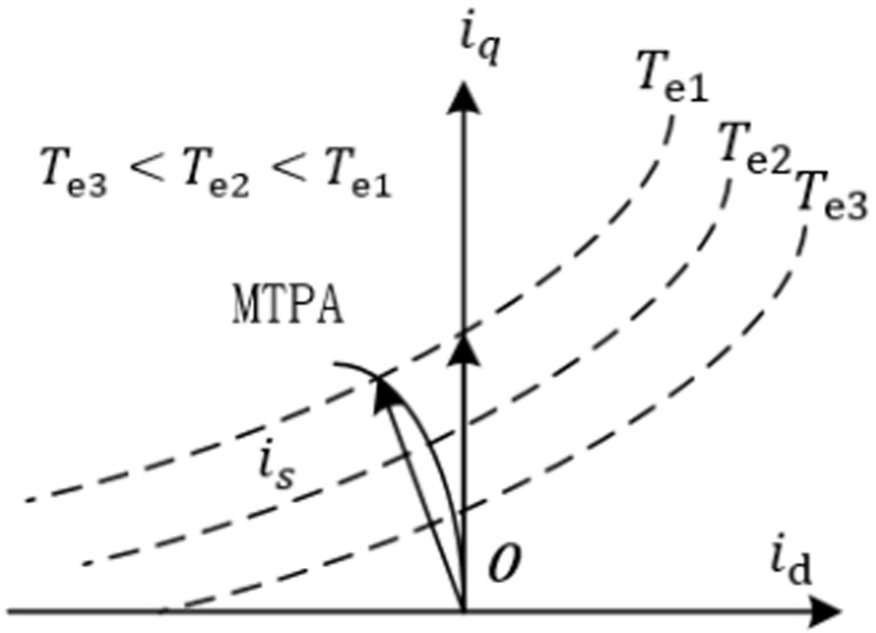
MTPA constant torque current variation curve.

Under the premise of stable motor operation, it can be observed that when the motor is given a specific setpoint, the quadrature-axis current is inversely proportional to the direct-axis current. Other factors, such as the inherent properties of the motor’s permanent magnets, are not considered for now. The trajectory of current variation along the d-q axes for constant torque is plotted, as shown in Fig 3.

From the above curve, it can be seen that there are three constant torque curves, denoted as $T_{\left\{e1\right\}}$, $T_{\left\{e2\right\}}$, and $T_{\left\{e3\right\}}$, with $T_{\left\{e1\right\}}>T_{\left\{e2\right\}}>T_{\left\{e3\right\}}$. At the moment when $\left(I_{d}=0\right)$ control is applied, the electromagnetic current curve is $i_{\left\{s2\right\}}$; however, if the MTPA control strategy is used, the current curve is $i_{\left\{s1\right\}}$. Clearly, under constant torque conditions, the current utilization of the MTPA control strategy is superior to the $\left(I_{d}=0\right)$ strategy. This is because the MTPA strategy can effectively adjust the current, requiring less current for a given torque, thus reducing the system’s energy consumption and losses.

Common methods for calculating the optimal matching of MTPA quadrature-axis and direct-axis currents include the formula calculation method, curve fitting method, and lookup table method, among others. This study will focus on the formula calculation method. Additionally, to seamlessly connect with the flux-weakening speed extension discussed later, the concept of the lead angle is introduced in advance.

Based on the control principle of MTPA (Maximum Torque Per Ampere), the maximum torque-to-current ratio is calculated by finding the extremum of the electromagnetic torque expression.

Introducing the stator current magnitude $i_{s}$:


$${\text{i}}_{\text{s}}\text{=}\sqrt{{\text{i}}_{\text{d}}^{\text{2}}{\text{+i}}_{\text{q}}^{\text{2}}}$$ (6)

$$\{\begin{array}{c} {\text{i}}_{\text{d}}{\text{=i}}_{\text{s}}\text{cosγ}\\ {\text{i}}_{\text{q}}{\text{=i}}_{\text{s}}\text{sinγ} \end{array}$$ (7)

To facilitate the calculation, the Lagrange multiplier λ is introduced, and an auxiliary function is constructed as follows:


$$\text{f=}\sqrt{{\text{i}}_{\text{d}}^{\text{2}}{\text{+i}}_{\text{q}}^{\text{2}}}\text{+λ}\left\{{\text{T}}_{\text{e}}\text{-}\frac{\text{3}}{\text{2}}{\text{P}}_{\text{n}}\left[{\text{ψ}}_{\text{f}}{\text{i}}_{\text{q}}\text{+}\left({\text{L}}_{\text{d}}{\text{-L}}_{\text{q}}\right){\text{i}}_{\text{q}}{\text{i}}_{\text{q}}\right]\right\}$$ (8)

Taking the partial derivative of the above expression and setting it to zero, we obtain:


$$\{\begin{array}{c} \frac{\partial \text{f}}{\partial {\text{i}}_{\text{d}}}\text{=}\frac{{\text{i}}_{\text{d}}}{\sqrt{{\text{i}}_{\text{d}}^{\text{2}}{\text{+i}}_{\text{q}}^{\text{2}}}}\text{-}\frac{\text{3}}{\text{2}}{\text{λP}}_{\text{n}}\left({\text{L}}_{\text{d}}{\text{-L}}_{\text{q}}\right){\text{i}}_{\text{q}}\text{=0}\\ \frac{\partial \text{f}}{\partial {\text{i}}_{\text{q}}}\text{=}\frac{{\text{i}}_{\text{q}}}{\sqrt{{\text{i}}_{\text{d}}^{\text{2}}{\text{+i}}_{\text{q}}^{\text{2}}}}\text{-}\frac{\text{3}}{\text{2}}{\text{λP}}_{\text{n}}\left[\left({\text{L}}_{\text{d}}{\text{-L}}_{\text{q}}\right){\text{i}}_{\text{d}}{\text{+ψ}}_{\text{f}}\right]\text{=0}\\ \frac{\partial \text{f}}{\partial \text{λ}}{\text{=T}}_{\text{e}}\text{-}\frac{\text{3}}{\text{2}}{\text{P}}_{\text{n}}\left[{\text{ψ}}_{\text{f}}\text{+}\left({\text{L}}_{\text{d}}{\text{-L}}_{\text{q}}\right){\text{i}}_{\text{d}}\right]{\text{i}}_{\text{q}}\text{=0} \end{array}$$ (9)

It can be computed that:


$${\text{i}}_{\text{q}}\text{=}\sqrt{\frac{{\text{ψ}}_{\text{f}}{\text{i}}_{\text{d}}}{\left({\text{L}}_{\text{d}}{\text{-L}}_{\text{q}}\right)}{\text{+i}}_{\text{d}}^{\text{2}}}$$ (10)

$${\text{i}}_{\text{d}}\text{=-}\frac{{\text{ψ}}_{\text{f}}}{\text{2}\left({\text{L}}_{\text{d}}{\text{-L}}_{\text{q}}\right)}\text{+}\sqrt{\frac{{\text{ψ}}_{\text{f}}^{\text{2}}}{\text{4}{\left({\text{L}}_{\text{q}}{\text{-L}}_{\text{d}}\right)}^{\text{2}}}{\text{+i}}_{\text{q}}^{\text{2}}}\text{=}\frac{{\text{-ψ}}_{\text{f}}\text{+}\sqrt{{\text{ψ}}_{\text{f}}^{\text{2}}{\text{+4i}}_{\text{q}}^{\text{2}}{\left({\text{L}}_{\text{d}}{\text{-L}}_{\text{q}}\right)}^{\text{2}}}}{\text{2}\left({\text{L}}_{\text{d}}{\text{-L}}_{\text{q}}\right)}$$ (11)

By solving Equations (4), (10), and (11) simultaneously, we obtain the functional relationship between the stator currents $i_{d}$, $i_{q}$, and the electromagnetic torque $T_{e}$:


$${\text{T}}_{\text{e}}\text{=}\frac{\text{3}}{\text{4}}{\text{pi}}_{\text{q}}\left[\sqrt{{\text{ψ}}_{\text{f}}^{\text{2}}\text{+4}{\left({\text{L}}_{\text{d}}{\text{-L}}_{\text{q}}\right)}^{\text{2}}{\text{i}}_{\text{q}}^{\text{2}}}{\text{+ψ}}_{\text{f}}\right]$$

$${\text{i}}_{\text{q}}\text{=}\sqrt[{\text{4}}]{\frac{\frac{\text{4}}{\text{9}}{\text{T}}_{\text{e}}^{\text{2}}\text{-}\frac{\text{2}}{\text{3}}{\text{T}}_{\text{e}}{\text{ψ}}_{\text{f}}{\text{i}}_{\text{q}}\text{p}}{{\text{p}}^{\text{2}}{\left({\text{L}}_{\text{d}}{\text{-L}}_{\text{q}}\right)}^{\text{2}}}}$$ (12)

The derivation of the above formula is based on the MTPA (Maximum Torque Per Ampere) method. As seen from Equations (11) and (12), this method involves multiple square root calculations and high-order multiplication operations, which place high demands on computer performance during simulation. Additionally, during weak magnetic flux expansion, it continues to produce highly complex calculations. Therefore, although this mathematical computation method is feasible, it has limitations in practical applications. Here, we introduce the advanced angle calculation MTPA to address these issues.

#### Calculation and control analysis of MTPA formula.

**Fig 4:**
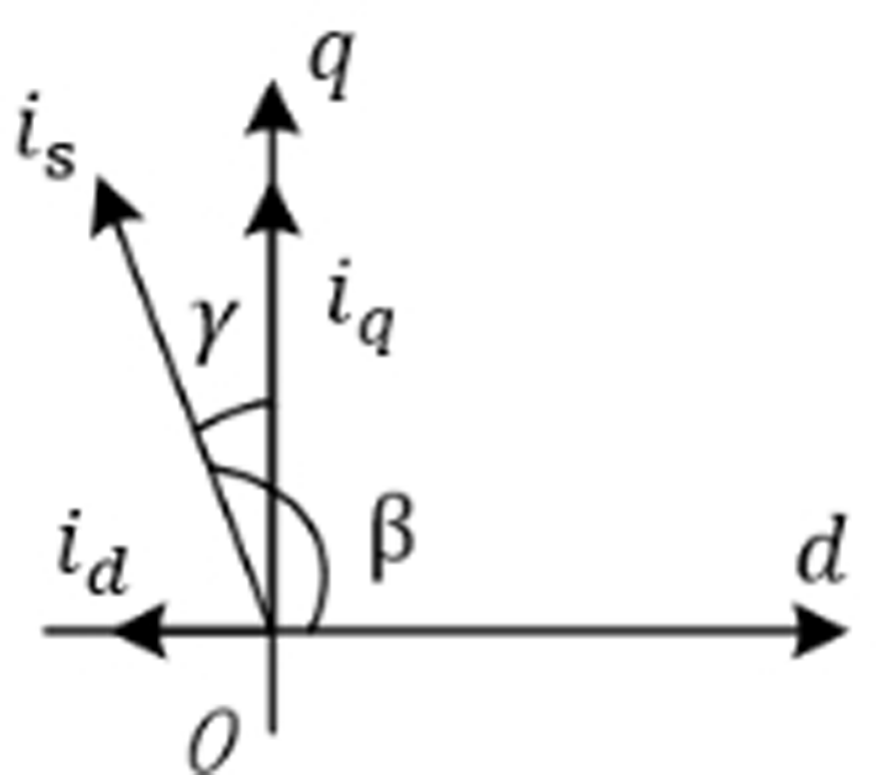
Stator current vector decomposition (See the figure file for details).


The common methods for calculating the optimal matching of MTPA cross-axis current include formula calculation method, curve fitting method, table lookup method, etc. This topic introduces the definition of lead Angle in order to reasonably connect with the following weak magnetic expansion velocity. As shown in the Fig 4, γ is the Angle between the intersection axis and the current amplitude, which is called the lead Angle, and the following expression can be obtained.

$$\gamma =\arccos\left(\frac{-\psi_{f}+\sqrt{\psi_{f}^{2}+8{\left(L_{d}-L_{q}\right)}^{2}i_{s}^{2}}}{4\left(L_{d}-L_{q}\right)i_{s}}\right)$$ (13)

Calculated:


$$\{\begin{array}{c} i_{d}=\frac{-\psi_{f}+\sqrt{\psi_{f}^{2}+8{\left(L_{d}-L_{q}\right)}^{2}i_{s}^{2}}}{4\left(L_{d}-L_{q}\right)}\\ i_{q}=\sqrt{i_{s}^{2}-i_{d}^{2}} \end{array}$$ (14)

MTPA advance Angle formula abandons the method of optimal distribution of alternating current and direct axis current by giving electromagnetic torque in complex mathematical model, instead, the optimal distribution of alternating current is carried out by giving stator current amplitude. In this method, the advance Angle γ value of the motor under working state is obtained by mathematical method, and the given value of the quadrature axis current and the given value of the direct axis current are optimally allocated by Equation (14).

**Fig 5:**
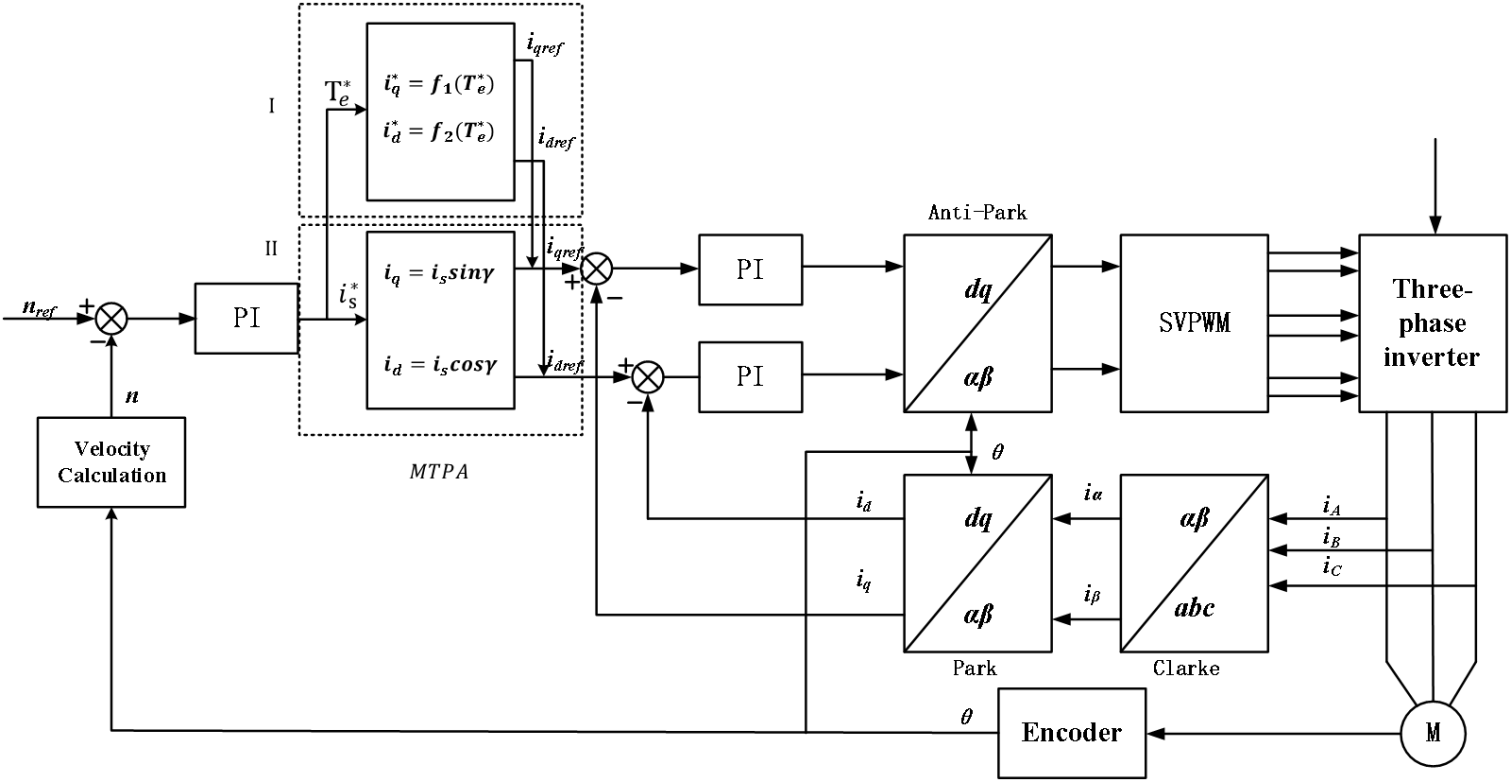
MTPA strategy motor control block diagram.

Fig 5 is the MTPA strategy motor control block diagram. In the figure, region I adopts the formula method of given electromagnetic torque to optimally distribute the quadrative-axis current and direct-axis current by given electromagnetic torque. In the II region, the strategy of optimal distribution of alternating direct axis current is adopted with the given stator current amplitude. Overall, the control block diagram is similar to the vector control block diagram, the given speed and the feedback speed are differentially fed into the speed PI controller. Subsequently, the output of the PI controller is a given electromagnetic torque/stator current amplitude, which is used to distribute the current. Then, the signal enters the current loop (the rest of the process is the same as the vector control system to achieve accurate control and regulation of the permanent magnet synchronous motor.

### Weak magnetic control

#### Weak magnetic control principle.

The MTPA control strategy is adopted for the electric motorcycle below the base speed. In order to realize the efficient operation of the electric motorcycle in a wide speed range, the weak magnetic control strategy is adopted to realize the operation above the base speed. With the rise of the motor speed, the motor back electromotive force will become larger and larger, and the inverter voltage has the limitation of voltage and current, so the motor cannot continue to increase the speed. In order to achieve a wide range of PMSM speed regulation.

Different from the traditional excitation motor, the PMSM rotor is the magnetic field generated by the permanent magnet, and the excitation current is not adjustable. The principle of weak magnetic control is to change the stator current to weaken the magnetic field generated by the permanent magnet under the limitation of the inverter voltage and current.

Therefore, the voltage and current of PMSM should meet the following limitations:


$$\{\begin{array}{c} u_{s}^{2}=u_{d}^{2}+u_{q}^{2}\leq u_{smax}^{2}\\ i_{s}^{2}=i_{d}^{2}+i_{q}^{2}\leq i_{smax}^{2} \end{array}$$ (15)

Where: $u_{d}$ and $u_{q}$ are the d-q axis voltage respectively; $i_{d}$ and iq are d-q axis current respectively, $u_{smax}$ is the maximum stator voltage and $u_{smax}=u_{dc}/\sqrt{3}$; i_smax indicates the maximum stator current.

When the motor is running stably, the current differential terms $\frac{d}{dt}i_{d}$ and $\frac{d}{dt}i_{q}$ can be approximately 0 and can be ignored. In addition, when the motor is running at high speed, the stator resistance voltage drop $Ri_{d}$ and $Ri_{q}$ relative to the back electromotive force can also be ignored. Then the stator voltage equation of the motor at high speed and stable operation:


$$\{\begin{array}{c} u_{d}=-{\text{ω}}_{e}L_{q}i_{q}\\ u_{q}=\omega_{e}\left(L_{d}i_{d}+\psi_{f}\right) \end{array}$$ (16)

By substituting formula (16) into formula (15) and sorting it out, we can get:


$${\left(\frac{i_{d}+\frac{\psi_{f}}{L_{d}}}{\frac{u_{smax}}{\omega_{e}L_{d}}}\right)}^{2}+{\left(\frac{i_{q}}{\frac{u_{smax}}{\omega_{e}L_{q}}}\right)}^{2}\leq 1$$ (17)

From the above formula, it can be seen that the voltage constraint equation of the motor is an ellipse with the center of the circle being ($-\frac{\psi_{f}}{L_{d}}$, 0). From the formula (16), it can be seen that the current constraint equation is a circle with the center of the circle being the coordinate origin (0, 0). The voltage limit ellipse and current limit circle are drawn under the d-q axis, as shown in Fig 4.

**Fig 6:**
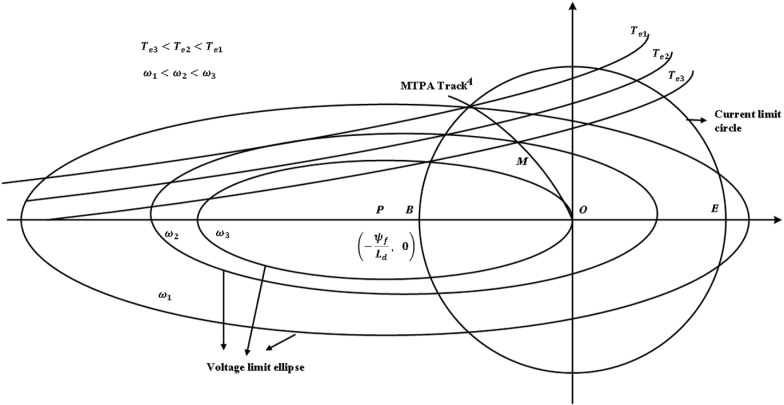
Voltage limit ellipse and current limit circle.

**Fig 7:**
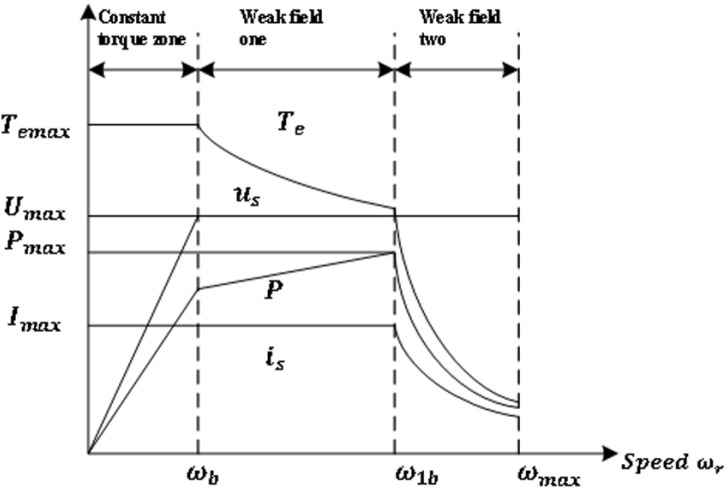
PMSM full speed operation area.

The full speed range of the motor can usually be divided into three operating regions: constant torque region (region I), weak magnetic region I (Region II), and weak magnetic region II (Region III), as shown in Fig 5. When the motor running speed is below the base speed, the inverter voltage is not limited, there is only a limit of current. At this time, the motor is in the constant torque zone, and the control strategy adopts maximum torque-current ratio control (MTPA). The current running curve is shown in Fig 6. Voltage limit ellipse and current limit circle O-A Fig 7.

**Fig 8:**
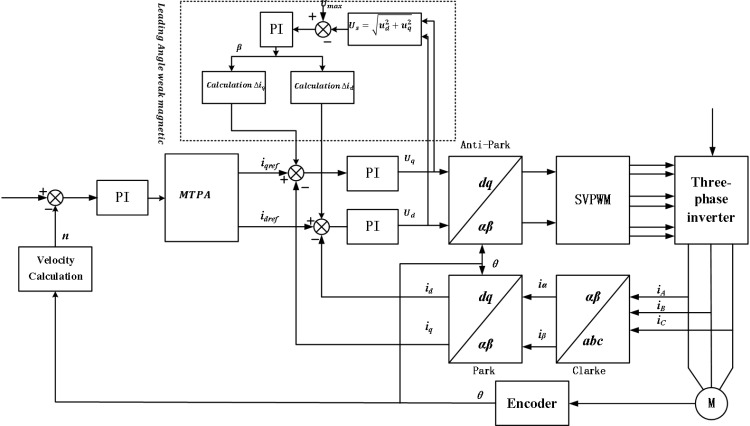
Block diagram of leading Angle weak magnetic control system.


When the motor speed is raised above the base speed, the inverter reaches the voltage and current limit of formula (15). At this time, it is necessary to carry out weak magnetic control to weaken the motor magnetic field, increase the motor speed, and move the motor operation area from the constant torque area to the weak magnetic zone I (zone II) control. In Fig 8, motor torque $T_{e3}<T_{e2}<T_{e1}$, motor speed $\omega_{1}<\omega_{2}<\omega_{3}$, when the motor speed rises from $\omega_{1}$ to $\omega_{2}$, the voltage limit ellipse will shrink in, and the original current MTPA operating curve O-A will become O-M. At this time, it is still controlled by MTPA, and its electromagnetic torque is reduced from $T_{e1}$ to $T_{e3}$, due to the control of the voltage limit circle, the current regulator reaches saturation at this time, and if the motor continues to increase speed, the motor will not be controlled, and the speed and other data will divergence. At this time, the motor speed cannot be accelerated by the voltage and current limit control of the inverter. If the M point is shifted to the N point, the current vector $i_{s}$ moves to the d axis, and the amplitude of the component $i_{d}$ on the d axis increases, which continues to be controlled by the voltage and current limit circle. At this time, the reverse direct axis magnetic field $L_{d}i_{d}$ generated by the direct axis current component can offset the permanent magnet magnetic field $\psi_{f}$. At the same time, the alternating axis current $i_{q}$ decreases, that is, the generated magnetic field $L_{q}i_{q}$ decreases, the weak magnetic acceleration is realized, the current regulator is out of saturation, the current control is restored, and the motor can continue to increase speed. This process is a weak magnetic acceleration process. It is represented by the following formula (18).

$${\text{ω}}_{e}=\frac{u_{smax}}{\sqrt{{\left(L_{q}i_{q}\right)}^{2}+{\left(L_{d}i_{d}+{\text{ψ}}_{f}\right)}^{2}}}$$ (18)

As shown in Fig 5, the decomposition of stator current vector, γ=0 when MTPA is controlled below the base speed, when the weak magnetic acceleration is carried out, the current vector $i_{s}$ moves to the d axis, at this time 0<γ<π/2, the D-axis current increases, and the Q-axis current decreases, and the process control of Equation (18) is realized. This method is advanced Angle weak magnetic control [19].

Weak magnetic two region is not considered in this project.

#### Leading angle weak magnetic control strategy.

The block diagram of the leading Angle weak magnetic control system is shown in Fig 8. First, it is necessary to determine whether the motor speed has reached the critical value by comparing the terminal voltage $U_{max}$ the inverter with the voltage square sum $\sqrt{u_{d}^{2}+u_{q}^{2}}$ of the current PI regulator. Secondly, through the voltage regulator, the Angle mapping is carried out to calculate the lead Angle γ of the motor speed change. PI regulation is divided into two stages.

$U_{max}-\sqrt{u_{d}^{2}+u_{q}^{2}}>0:$ voltage did not reach the maximum limiting, the motor speed did not enter the rated speed state (below the base speed), at this time the voltage regulation is positive, due to the limiting effect, the voltage loop output value γ=0, at this time the motor has not yet entered the weak magnetic state.

$U_{max}-\sqrt{u_{d}^{2}+u_{q}^{2}}<0:$ voltage reaches the maximum value, at this time the motor speed is above the base speed, the voltage loop PI output value γ<0, at this time the motor runs into a weak magnetic state. Leading Angle d, q axle load new distribution current calculation formula:


$$\{\begin{array}{c} i_{d}=i_{s}sin\gamma \\ i_{q}=i_{s}cos\gamma \end{array}$$ (19)

## Control strategy algorithm simulation design

### MTPA module construction

**Fig 9:**
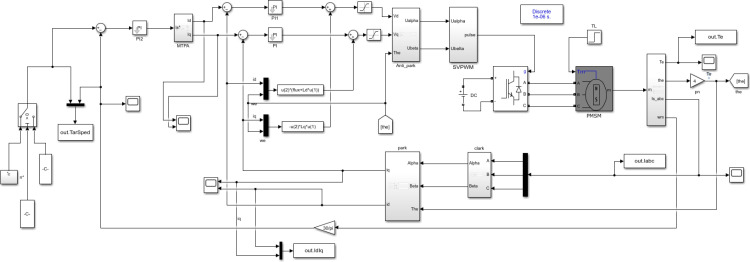
PMSM vector model based on MTPA control strategy.

Compared with vector control module, MTPA strategy introduces a special module for distributing stator current. Fig 9 shows the PMSM model controlled by MTPA built on the SIMULINK platform.

**Fig 10:**
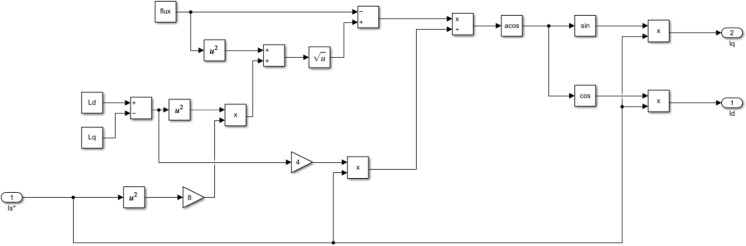
Internal module of MTPA algorithm.

Fig 10 is the MTPA algorithm module. The module uses the output value of the speed PI regulator as the given stator current amplitude, and uses formula (2) to calculate the lead Angle γ. Finally, according to the relationship between $i_{d}=i_{s}$cosγ and $i_{q}$ = $i_{s}\sin\gamma$, the D-axis and Q-axis currents are allocated to achieve vector control.

#### Establishment of leading Angle weak magnetic control module.

**Fig 11:**
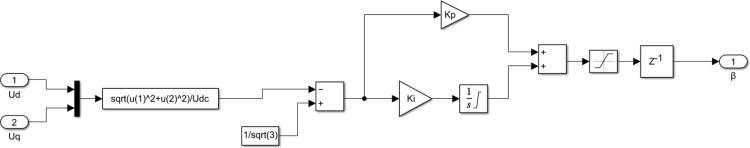
Internal modules of the weak magnetic control strategy.

To achieve weak magnetic control above the base speed, it is first necessary to determine whether the speed threshold is reached and enter the weak magnetic control, and then the voltage PI control is used to map the weak magnetic advance Angle, and then the stator current is distributed by the formula Fig 11.

**Fig 12:**
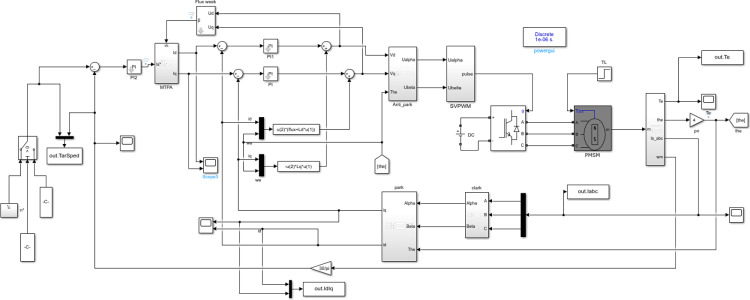
PMSM vector model based on MTPA+ leading Angle weak magnetic control strategy.

In addition, to realize the full speed range control of the motor, in addition to the base speed above the base speed should also consider the base speed below, so the model built in this topic is MTPA+ weak magnetic vector control of leading Angle. Fig 12 Different from Fig 10, in the internal module of MTPA, it is necessary to determine which control strategy to execute according to the weak magnetic lead Angle. The solution in this paper is to use MATLAB C-function module to write C code for smooth conversion of control strategy.

### Introduction to the APP interface

#### Interface introduction.

**Fig 13:**
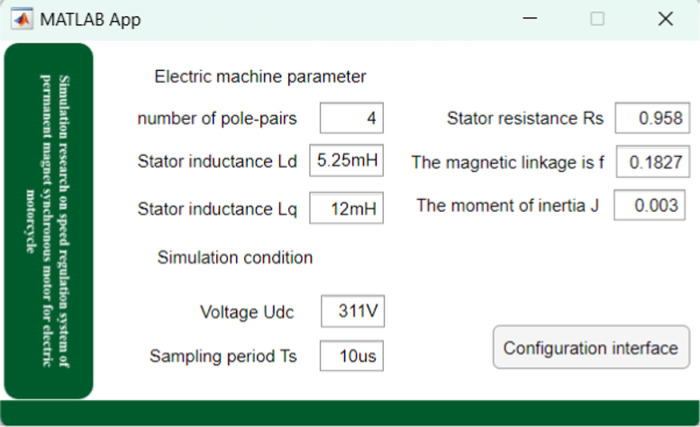
Display interface of system motor parameters.

Fig 13 shows the system motor parameter display interface, the upper part of the interface shows the school emblem of Jiamusi University, and the middle part shows the project name: “Simulation Research on Permanent magnet synchronous Motor Speed Regulation System of Electric Motorcycle”. The lower part lists the parameters of the motor and includes an interface switch button.

**Fig 14:**
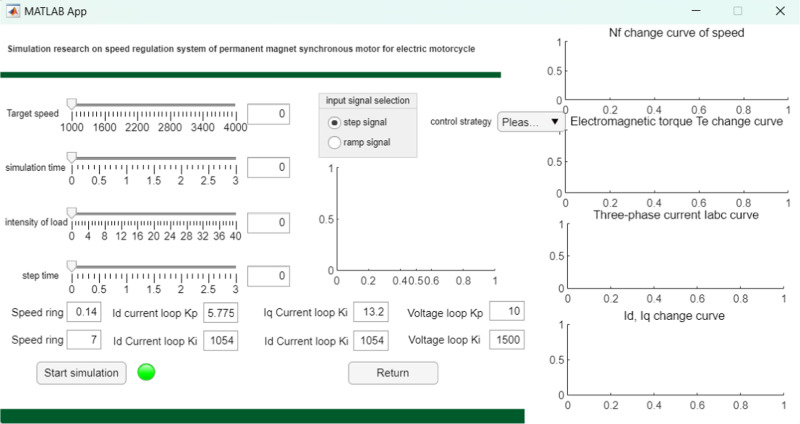
System operation control interface.

Fig 14 System operation control interface. The left half is the data control of target speed, simulation time, load strength and step time. The data is set by slider control and displayed by text control. The middle part of the interface includes input signal selection and control strategy selection. The input signal selection uses radio button group controls, including step signals and ramp signals, and displays the signal curve by axis. The control policy is selected through the drop-down box control, including Id=0, MTPA, weak magnetic control policy. The lower left part of the interface is the parameter setting part of the motor PI regulator, whose parameter values have been calculated and the simulation system has been given. In addition, there are two button controls on the interface, one for starting the simulation and the other for going back to the home page to switch the interface. On the right side of the interface, the motor simulation results are displayed, including the changes of speed $N_{f}$, electromagnetic torque $T_{e},$ three-phase current $i_{abc}$ and current $i_{d}$ and $i_{q}.$ The coordinate image is displayed when the simulation runs until the end time.

## System simulation model analysis and debugging

### Basic system parameters

In order to verify the operation of electric motorcycle PMSM in the full speed range, the motor model as shown in Fig 7 was built in MATLAB/SIMULINK environment, and the basic parameters of PMSM were given as shown in Table 1.

**Table 1:** PMSM parameters.

Parameter name	Value
Motor structure	IPMSM
dc voltage $U_{\left\{dc\right\}}$	311 V
Number of poles $p_{n}$	4
Stator inductance $L_{d}$	5.25 mH
Stator inductance $L_{p}$	12 mH
Stator resistance $R_{s}$	0.958 *Ω*
Magnetic linkage	0.1827 *Wb*
The moment of inertia J	0.003 kg·m^3^
Damping coefficient B	0.008 N·m·s

In addition to the inherent attribute parameters of PMSM itself, the proportional integral coefficient of PI regulator is needed to calculate the proportional integral coefficient of speed PI regulator and current PI regulator, as shown in Table 2 below.

**Table 2:** Coefficient values of each PI regulator.

Parameter name	Value	Parameter name	Value	Parameter name	Value	Parameter name	Value
*Speed ring* $K_{p}$	0.14	$I_{d}CurrentloopK_{p}$	5.775	$I_{q}CurrentloopK_{p}$	13.2	$VoltageloopK_{p}$	10
*Speed ring* $K_{i}$	7	$I_{d}CurrentloopK_{i}$	1054	$I_{q}CurrentloopK_{i}$	1054	$VoltageloopK_{i}$	1500

### Experimental setup

Load the above data into each simulation file and start the simulation.

Experiment (1), motor operation experiment below base speed, given the step signal, the motor speed jumped from 0 to 1000r/min, the initial load of the motor was 5 N∙m, the load torque changed to 10 N∙m at 0.5s, and then the motor speed always maintained 1000r/min steady state operation. Set the simulation time to 1.2s.

Experiment (2), motor operation experiment below base speed, given slope signal, motor target speed 1500r/min, the initial load of the motor is 5 N∙m, the load torque changes to 10 N∙m at 0.5s, and then the motor speed always maintains 1500r/min steady state operation. Set the simulation time to 1.2s.

Experiment (3), motor operation experiment above base speed, given the step signal, respectively verify Id = 0, MTPA, weak magnetic control speed regulation strategy for rated speed limit test, initial load torque is 5 N∙m.

Experiment (4), motor operation experiment above base speed, given the step signal, motor speed from 0 to 4000r/min, the initial load torque of the motor is 5 N∙m, the load torque changes to 10 N∙m at 0.5s, and then the motor speed always maintains 4000r/min steady state operation.

### Experimental verification

**Fig 15:**
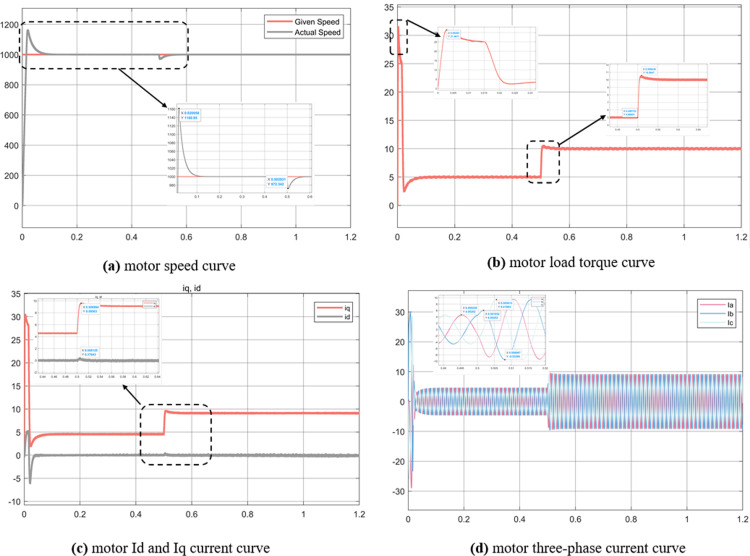
Simulation results of the control strategy of experiment 1 Id = 0.

**Fig 16:**
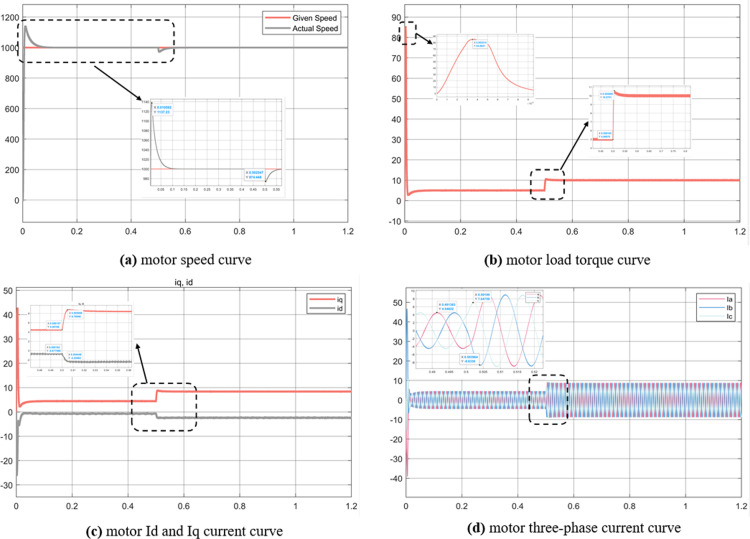
Simulation results of experiment 1 MTPA control strategy.

The simulation results of experiment 1 are shown in Figs 15 and 16.

It can be seen from Figs 15 and 16 that under the step signal, the MTPA control strategy speed rise time is as fast as 0.01s to reach the maximum overshoot value of 1137 r/min, the steady speed reaches 1000 r/min at 0.09s, and the steady speed reaches the steady state after the load disturbance at 0.56s. The overshoot value of the load disturbance speed is 970 r/min. The strategy with Id = 0 reaches the maximum overshoot value of 1161 r/min at 0.02, the steady speed of 1000 r/min at 0.13s, and the steady speed after the load disturbance at 0.57s, and the overshoot value of the load disturbance speed is 975 r/min. From the speed curve, MTPA overshoot is small and has a fast adjustment time. It can be seen from Figure (b) that MTPA’s load torque reaches 85 N∙m when the motor starts, and the overshoot is almost 0 when the load changes abruptly. The starting torque of MTPA is 32 N∙m when Id = 0, and the load sudden overshoot is 0.5 N∙m. This curve shows that the MTPA control strategy rapidly increases the torque at start-up to distribute stator current to achieve rapid motor speed following.

Figure (c) shows the current curves of Id and Iq. The starting current of the two control strategies is 30A and 42A respectively, which is consistent with the conclusion of load torque. When steady state is reached, Id = 0 strategy, Iq = 9.36A, Id = 0A, and MTPA strategy Iq = 8.78A, Id = -2.54A. Because MTPA assigns stator current Id, Iq decreases and has a weak magnetic effect. Figure (d) is a three-phase current curve, and the conclusion is basically consistent with Figure (c).

**Fig 17:**
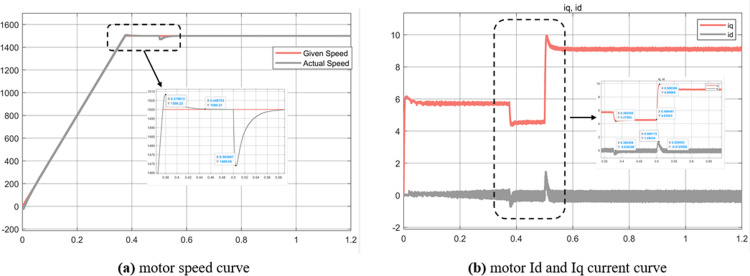
Simulation results of control strategy in experiment 2 with Id = 0.

**Fig 18:**
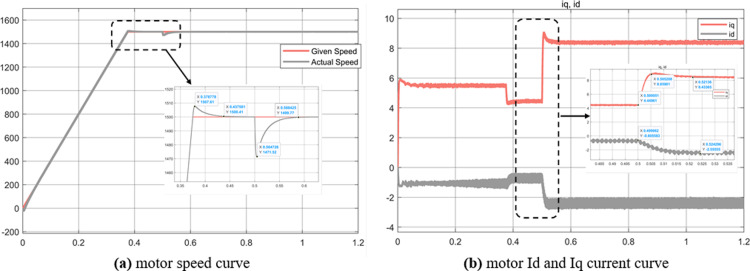
Simulation results of MTPA control strategy in experiment 2.

Experiment (2): From the comparison of Figs 17 and 18, it can be concluded that there is no significant difference between the motor speed curve under the step signal and the speed curve under the step signal. When the MTPA speed reaches 1500 r/min, the speed overshoot is 1507r/min, and the Id = 0 control strategy reaches 1510r/min. When a load change of 10 N∙m is received at 0.5s, the MTPA overshoot is still lower than the Id = 0 control strategy, and the adjustment time is faster. Under the Id and Iq current curves, the Id current of the former is 0, the Iq overshoot current is 10A, the steady-state current is 9A, and the MT`PA strategy Iq overshoot current is 9A, the steady-state current is 8.5A, and the Id current is -2.25A. Under the same electromagnetic torque, the MTPA current distribution strategy is better.

**Fig 19:**
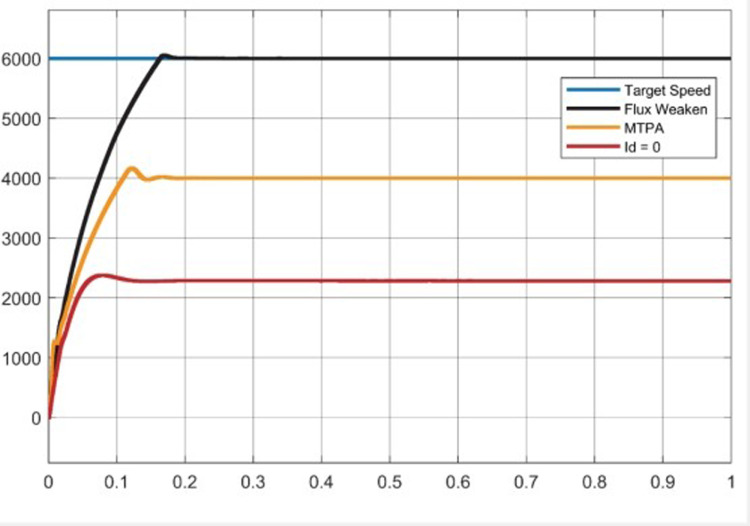
Experiment 3 Motor running speed upper limit curve.

Experiment (3): Fig 19 represents the test curve of the motor speed limit of the three control strategies. Given the target speed of 6000 r/min, the maximum speed of the three control strategies of Id = 0, MTPA and weak magnetic field are respectively: 2361 r/min, 4163 r/min, 6030 r/min, and can continue to the end of the simulation. In addition, it can be seen from the image that the rise time of the three control strategies increases successively, which is 0.07s, 0.12s, 0.16s, respectively. In the motor full speed operation range, weak magnetic control can reach the base speed above, MTPA is often used for base speed below the control, its speed expansion ability is weak, Id = 0 speed expansion ability is weaker, only applicable to the base speed below.

**Fig 20:**
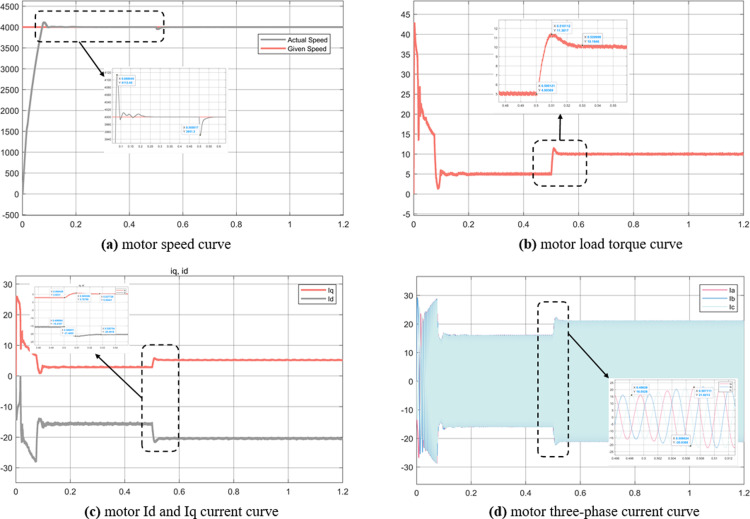
Simulation results of magnetic weakness control strategy in experiment 4.

Experiment (4): It can be seen from Fig 20 that the weak magnetic control can still run stably when the speed is 4000r/min, the overshoot is 110, and the adjustment time is fast. In addition, when the load changes at 0.5s, it can still be quickly adjusted, with good stability and robustness. In Figure (b), it can be seen that the motor torque changes greatly when the motor is started, which is related to the MTPA control strategy adopted below the motor base speed. It can be seen from Figure (c) that the stator current Id allocated by the weak magnetic control algorithm is very large -20A, and the Iq is 5.1A, which is different from the simulation results of experiment 1 MTPA control strategy. From this data, it can be seen that the current allocated by the weak magnetic field Id is larger, so as to offset the magnetic field generated by the permanent magnet to achieve weak magnetic acceleration.

## Conclusion and future directions

In conclusion, this work successfully presents a high-performance, low-cost electric motorcycle controller that integrates PMSM’s dynamic control strategies, improving both motor efficiency and overall vehicle performance. The main achievements include:

1. Development of dynamic control algorithms for PMSM, including Id = 0, MTPA, and flux-weakening strategies, enhancing motor performance across various speed and load conditions.
2. Real-time dynamic switching between MTPA and flux-weakening control, expanding the PMSM’s speed range and providing smooth operation during acceleration.
3. Simulation results show significant improvements in response speed, efficiency, and anti-interference performance compared to existing control methods.

However, the study has limitations in practical implementation, and the next steps will involve the following:

Future work will focus on implementing the proposed controller on a real electric motorcycle, conducting experimental tests to verify the simulation results under real-world conditions. The control algorithms can be further refined to account for factors such as system aging, power losses, and varying environmental conditions. Long-term field testing is necessary to validate system performance, robustness, and durability over extended operational periods.

### What is the advantage over established techniques

Enhanced Control Flexibility: The dynamic working region optimization adjusts the control algorithm in real-time, ensuring efficient torque output and energy utilization across varying speeds and load conditions. This provides better adaptability compared to traditional control strategies.

Smooth Transition between MTPA and Flux-Weakening Control: This method ensures smooth switching between MTPA and flux-weakening control strategies, effectively expanding the operational range of the PMSM and improving performance at both low and high speeds. It addresses the limitations of traditional fixed strategy switching.

Cost-Effective and Efficient Design: The proposed MTPA control strategy helps improve inverter efficiency, reducing costs and enhancing system performance during motor startup. Additionally, flux-weakening control enables a maximum speed of up to 6000 rpm, offering a significant advantage over Id = 0 or MTPA control strategies.

Strong Anti-Interference Performance: With current and speed PI controllers, the system demonstrates strong responsiveness and anti-interference capabilities, outperforming traditional designs in terms of stability and robustness.

## Supporting information

S1 Table PMSM parameters. (PDF)

S2 Table Coefficient values of each PI regulator. (PDF)
