# Supplementary material for: Research on simulation of permanent magnet synchronous motor in full speed range
Source: PLoS One. 2025 Apr 21;20(4):e0320786. doi: 10.1371/journal.pone.0320786 (PMC12011299; doi:10.1371/journal.pone.0320786)
Supplement: S1 Table — (PDF) [file pone.0320786.s001.pdf]

Parameter name	Value
Motor structure	IPMSM
dc voltage $U_{\{dc\}}$	311V
Number of poles p_n	4
Stator inductance L_d	5.25mH
Stator inductance L_p	12mH
Stator resistance R_s	0.958Ω
Magnetic linkage	0.1827Wb
The moment of inertia J	0.003 kg· m^3
Damping coefficient B	0.008N · m · s
