# Supplementary material for: Research on simulation of permanent magnet synchronous motor in full speed range
Source: PLoS One. 2025 Apr 21;20(4):e0320786. doi: 10.1371/journal.pone.0320786 (PMC12011299; doi:10.1371/journal.pone.0320786)
Supplement: S2 Table — (PDF) [file pone.0320786.s002.pdf]

Parameter Name	Value	Parameter Name	Value	Parameter Name	Value	Parameter Name	Value
Speed ring K_p	0.14	$I_d Currentloop K_p$	5.775	$I_q Currentloop K_p$	13.2	$Voltageloop K_p$	10
Speed ring K_i	7	$I_d Currentloop K_i$	1054	$I_q Currentloop K_i$	1054	$Voltageloop K_i$	1500
